# Medication use patterns among older patients in temporary stays in Denmark

**DOI:** 10.1007/s41999-025-01210-2

**Published:** 2025-05-07

**Authors:** Hanin Harbi, Carina Lundby, Peter Bjødstrup Jensen, Søren Post Larsen, Linda Grouleff Rørbæk, Lene Vestergaard Ravn-Nielsen, Jesper Ryg, Mette Reilev, Kasper Edwards, Anton Pottegård

**Affiliations:** 1 Clinical Pharmacology, Pharmacy and Environmental Medicine, Department of Public Health, University of Southern Denmark, Campusvej 55, 5230 Odense M, Denmark; 2 Hospital Pharmacy Funen, Odense University Hospital, Odense, Denmark; 3 REAGENS, Skårup Fyn, Denmark; 4 Komponent, Copenhagen, Denmark; 5 Geriatric Research Unit, Department of Clinical Research, University of Southern Denmark, Odense, Denmark; 6 Department of Geriatric Medicine, Odense University Hospital, Odense, Denmark; 7 Centre for Suicide Research, Odense, Denmark; 8 The Research Unit in Psychiatry - Child and Adults, Psychiatry in the Region of Southern Denmark, Aabenraa, Denmark; 9 DTU Engineering Technology, Technical University of Denmark, Ballerup, Denmark

**Keywords:** Epidemiology, Drug utilization, Polypharmacy, Intermediate care facilities, Skilled nursing facilities, Aged

## Abstract

**Purpose:**

Patients in temporary stays are typically older individuals with frailty and multimorbidity. However, limited knowledge exists about their medication use. This study aimed to describe prescription drug use among patients in temporary stays in Denmark.

**Methods:**

We conducted a drug utilisation study on 11,424 patients in public healthcare-operated temporary stay units across 14 Danish municipalities between 2016 and 2023 (median age 81 years; 54% women). Prescription data were sourced from the Danish National Prescription Registry.

**Results:**

Patients used a median of six drug classes (interquartile range [IQR] 4–10) in the four months before moving into a temporary stay facility; 68% used ≥ 5 drug classes, and 26% used ≥ 10. The most commonly used drug classes were paracetamol (49%), statins (30%), and proton pump inhibitors (29%). The monthly rate of new drug use increased from 23/100 patients six months before move-in to a peak of 262/100 patients in the first month after move-in, driven primarily by laxatives, analgesics, and antibiotics. High-risk drug use increased from 70 to 83% following move-in, with 49% of patients initiating at least one new high-risk drug, most commonly opioids (28%), potassium (17%), and anticoagulants and platelet inhibitors (15%). General practitioners initiated 60–70% of treatments and maintained 80–90%. Hospital physician prescriptions increased around move-in, peaking at 55% for initiation and 25% for maintenance in the first month after move-in.

**Conclusion:**

Patients in temporary stays in Denmark demonstrate high medication use, including high-risk drugs, with a notable increase in treatment initiations around the time of move-in.

**Supplementary Information:**

The online version contains supplementary material available at 10.1007/s41999-025-01210-2.

## Key summary points

**Aim:**

To describe medication use patterns among patients in temporary stay facilities in Denmark.

**Findings:**

Half of the patients used more than six drug classes when entering the facility, with a substantial use of high-risk drugs, which increased upon entry. Entry into a temporary stay facility was associated with a marked increase in the initiation of new treatments.

**Message:**

Patients in temporary stay facilities have high levels of polypharmacy, with treatment initiations increasing sharply upon entry.

**Supplementary Information:**

The online version contains supplementary material available at 10.1007/s41999-025-01210-2.

## Introduction

Healthcare systems worldwide are increasingly challenged by ageing populations and the growing burden of chronic diseases. Consequently, patients are being discharged from hospitals earlier, often in less stable condition. This shift has increased the demand for community-based care services to support earlier hospital discharges and prevent hospital (re)admissions [1–5]. One such service is temporary stay facilities, which provide short-term care outside the home, typically for patients who need additional support to recover and regain their strength after a hospital stay. These patients are often older individuals with frailty and multimorbidity. Medication use is substantial among these patients [6–11]. A recent study described the challenges of transitioning patients from hospital to temporary stay facilities, reporting a median use of 8 drugs per patient, with 96% using at least one high-risk drug [12].

Managing medications in temporary stay facilities poses several challenges for care staff. First, care staff do not have access to patients’ medical records. When patients are discharged from a hospital to a temporary stay facility, the care staff does only receive a discharge notice from the hospital nurses, rather than a detailed discharge letter from the hospital physician. Additionally, temporary stay facilities typically do not store medication. Instead, patients must either bring their own medications or have them delivered by a relative or pharmacy. These challenges force care staff to invest considerable time and resources in gathering information about patients’ medication and resolving any discrepancies [8, 12].

One study found that only half of the patients discharged from hospitals to temporary stay facilities arrived with all the necessary medication. As a result, nurses had to contact hospital physicians and general practitioners (GPs) repeatedly to clarify medication regimens. It was estimated that one-third of these contacts could have been avoided if the discharge letter had been provided [12].

To improve the treatment and safety of patients in temporary stays, a better understanding is needed not only of medication management challenges but also the quantity and types of medications being used, as well as any changes in medication use around the time of moving into a facility. Therefore, the aim of this study was to describe the use of prescription drugs among patients in temporary stays in Denmark.

## Methods

We established a cohort of 11,424 patients who stayed in temporary care facilities across 14 Danish municipalities during 2016 and 2023. The cohort was supplemented with individual-level data from the Danish National Prescription Registry.

### Data sources

Data on temporary stays were provided by the municipalities for all or part of the study period from January 1, 2016, to December 31, 2023. These data included each patient’s Central Person Register (CPR) number, move-in date, and move-out date. We linked this information to the Danish National Prescription Registry to obtain prescription drug use data. Individual-level linkage was achieved using the CPR number, a unique personal identifier assigned to all Danish residents by the Civil Registration System since 1968 [13].

The Danish National Prescription Registry includes data on all prescription drugs dispensed at Danish community pharmacies since 1995, categorized by the Anatomical Therapeutic Chemical (ATC) classification system. It also contains the dispensing date and prescriber identifier [14]. The prescriber identifier allowed us to categorise prescribers as GPs, private practicing specialists, or hospital physicians. The validity of prescriber data in the Danish National Prescription Registry is high and improving over time [15]. To identify the medical speciality of primary sector prescribers (GP or private practicing specialist), we linked the prescriber identifier to the Registry of Health Care Providers [16]. Data on patient death were obtained from the Civil Registration System [13].

### Study cohort

We included temporary stays if both the move-in and move-out dates occurred within the study period. Temporary stays with missing or invalid CPR numbers, move-in dates, or move-out dates were excluded, as were cases where the move-out date preceded the move-in date. We required patients to have resided in Denmark for at least 2 years prior to their first temporary stay. For patients with multiple temporary stays, we combined overlapping temporary stays into a single continuous stay. Temporary stays were considered overlapping if there was no gap between the move-out date of one stay and the move-in date of the next. After combining overlapping temporary stays, 21% (2422/11,424) of the patients had more than one temporary stay during the study period. Only the first temporary stay for each patient was included in the analyses to avoid difficulties in interpretation due to non-independent observations.

### Setting

Temporary stays are provided by Danish municipalities for individuals with short-term care and support needs that cannot be met at home. These stays are available to all eligible citizens in the municipality, with no limit on their duration. Access to temporary stays is managed by the municipality. The types of stays may vary between municipalities but can include care or rehabilitation after illness or hospitalisation, as well as respite for family caregivers. The care staff may include nurses, care assistants, physiotherapists, and occupational therapists, among others. Temporary stay facilities are not required to have physicians on staff. Instead, the primary medical responsibility lies with the patient’s general practitioner or the discharging hospital. The coverage of expenses for the stay depends on its type. The so-called “acute beds” are free of charge. These beds fall under the Danish Health Act and are subject to quality standards set by the Danish Health Authority, including the requirement for round-the-clock nursing care, and accommodate the most acutely ill and unstable patients. All other types of stays fall under the Danish Social Services Act and require a small co-payment for services such as meals and laundry [8].

### Analyses

First, we described baseline medication use as of the day the patient moved into the temporary stay facility. For each patient, we calculated the median number of drug classes (ATC level 4) dispensed in the four months prior to move-in. We also determined the proportion of patients using at least five drug classes (polypharmacy) and at least ten drug classes (excessive polypharmacy), defined as filling at least one prescription for five or ten different drug classes, respectively, within the 4-month period before move-in. Additionally, we identified the most frequently dispensed drug classes and calculated the proportion of patients filling at least one prescription from each main drug group, categorized by the first level of the ATC code (i.e., by target organ or system). The 4-month time window accounts for the typical prescription of 100 units in Denmark and allows for some degree of non-compliance and irregular dispensing.

Second, we analysed changes in medication use around the time of moving into the temporary stay facility, focusing on the 2 years before and after move-in. We chose this time period because temporary stays typically follow a period of health decline, which may lead to increased medical attention and, consequently, medication changes. Additionally, patients have a median survival of about 2 years after move-in [17]. We calculated the monthly rate of incident drug use per 100 patients. Incident use was defined as the first filled prescription for a drug class that had not been dispensed to the patient within the previous 2 years. We also identified the drug classes contributing to peaks in incident drug use.

Third, we assessed high-risk drug use at baseline. High-risk drugs were defined according to the Danish Patient Safety Authority and included anticoagulants and platelet inhibitors (ATC code B01A), antidiabetics (A10), digoxin (C01AA05), low-dose methotrexate (L04AX03), opioids (N02A and R05DA04), and potassium (A12B). These drugs are frequently associated with adverse drug events resulting from medication errors and require special attention from healthcare professionals [18]. We excluded gentamicin as it is rarely used outside of hospital settings due to the careful monitoring it requires. For each high-risk drug, we calculated the proportion of patients filling at least one prescription in the following periods: 1) 4 months before move-in, 2) 4 months after move-in, 3) before but not after, 4) after but not before, and 5) both periods.

Finally, we described the types of prescribers responsible for initiating treatment (incident prescriptions) and maintaining treatment (nonincident prescriptions, i.e., all prescriptions other than incident prescriptions). For each month in the 2 years before and after move-in, we calculated the proportion of incident and nonincident prescriptions issued by GPs, private practicing specialists, and hospital physicians.

All analyses were performed using R version 4.3.3.

## Results

We identified 11,424 patients who moved into a temporary stay facility during the study period, analysing only their first temporary stay. The median age of the patients at move-in was 81 years (interquartile range [IQR] 73–87 years), and 54% were women. The median Charlson Comorbidity Index score was 1 (IQR 0–2), and the median number of hospital admissions in the year prior to move-in was 3 (IQR 2–6) (Supplementary Table 1).

The patients used a median of 6 drug classes (IQR 4–10) in the 4 months prior to moving into the temporary stay facility. Polypharmacy (defined as using at least five drug classes) was observed in 68% of patients, while 26% of patients exhibited excessive polypharmacy (i.e., using at least ten drug classes). The most frequently used drug classes included paracetamol (49%), statins (30%), proton pump inhibitors (29%), platelet inhibitors (26%), and selective beta blockers (23%) (Table 1). The most commonly used drug groups were those related to the nervous system (72%), cardiovascular system (71%), alimentary tract and metabolism (59%), and blood and blood-forming organs (51%) (Supplementary Table 2).

**Table 1:** The 25 most frequently dispensed drug classes at time of moving into a temporary stay facility^a^

ATC code	Drug class	Proportion of prevalent users, n (%) (n = 11,424)
N02BE	Anilides	5588 (49)
C10AA	HMG CoA reductase inhibitors	3463 (30)
A02BC	Proton pump inhibitors	3274 (29)
B01AC	Platelet aggregation inhibitors excl. heparin	2995 (26)
C07AB	Beta blocking agents, selective	2628 (23)
C03CA	Sulfonamides, plain	2582 (23)
A12BA	Potassium	2478 (22)
C08CA	Dihydropyridine derivatives	2173 (19)
N02AA	Natural opium alkaloids	2001 (18)
B01AF	Direct factor Xa inhibitors	1752 (15)
J01CA	Penicillins with extended spectrum	1744 (15)
C09AA	ACE inhibitors, plain	1652 (14)
C09CA	Angiotensin II receptor blockers (ARBs), plain	1576 (14)
N06AB	Selective serotonin reuptake inhibitors	1389 (12)
N02AX	Other opioids	1292 (11)
N06AX	Other antidepressants	1177 (10)
A06AD	Osmotically acting laxatives	1160 (10)
A10BA	Biguanides	1076 (9.4)
R03AC	Selective beta-2 adrenoreceptor agonists	1056 (9.2)
H02AB	Glucocorticoids	1054 (9.2)
N05CF	Benzodiazepine related drugs	1045 (9.1)
N02BF	Gabapentinoids	1001 (8.8)
C03AB	Thiazides and potassium in combination	856 (7.5)
M01AE	Propionic acid derivatives	843 (7.4)
J01CE	Beta-lactamase sensitive penicillins	843 (7.4)

^a^Medication use at the day of moving into the temporary stay facility was determined by assessing filled prescriptions within 4 months prior to move-in

**Fig. 1:**
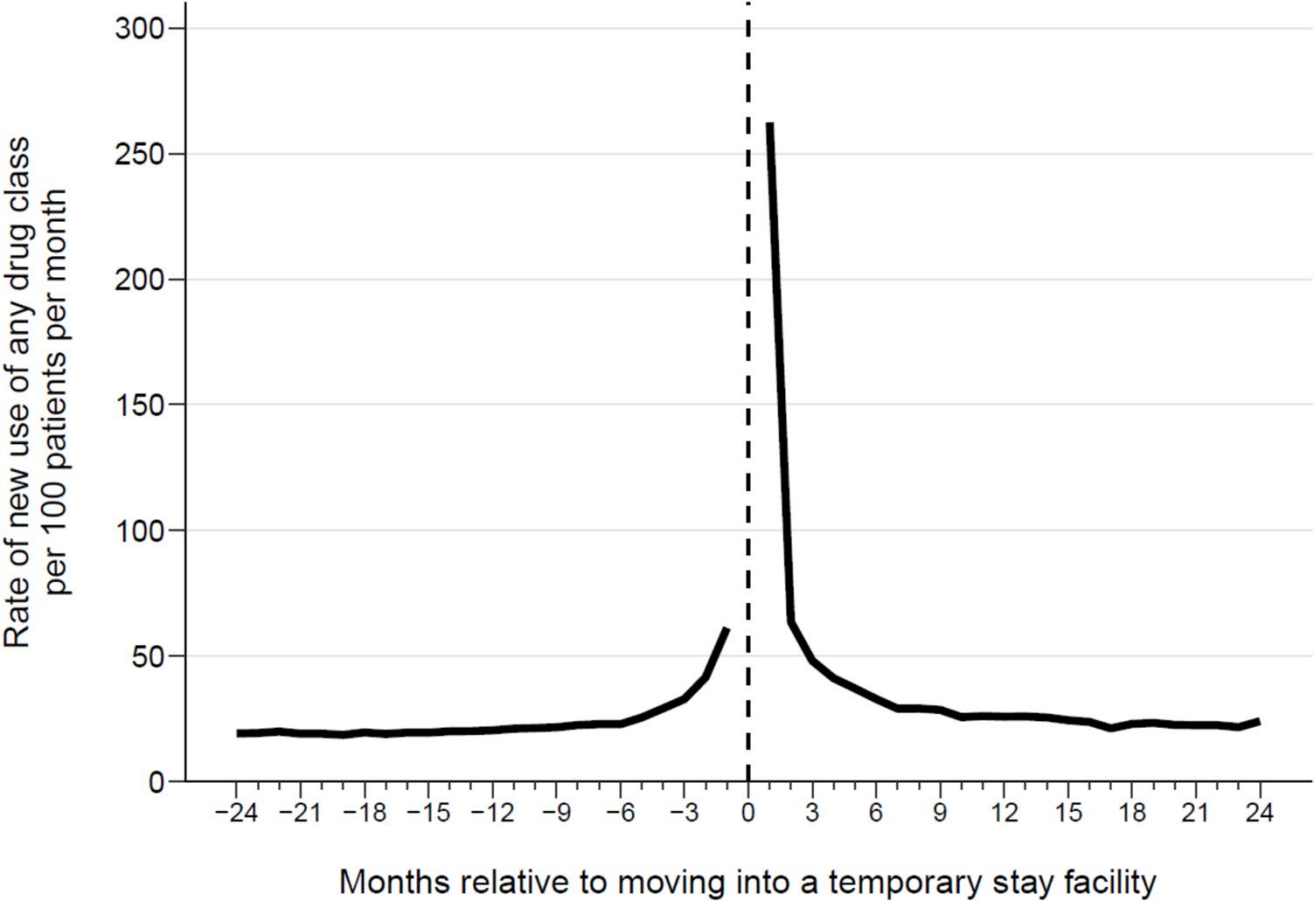
Monthly rate of incident drug use per 100 patients in the 2 years before and after moving into a temporary stay facility

The monthly rate of incident use increased from 23 per 100 patients six months prior to move-in to 61 per 100 patients in the month before move-in. The rate peaked at 262 per 100 patients per month in the month following move-in, then gradually decreased to a level slightly higher than premove-in around 9 months after the move (Fig. 1). The primary drug classes responsible for this peak in incident use were laxatives, analgesics, and antibiotics (Supplementary Table 3, 4, and 5).

The proportion of patients using at least one high-risk drug increased from 70% in the four months before move-in to 83% in the 4 months following move-in. In both periods, the most frequently used high-risk drugs were anticoagulants and platelet inhibitors (46% before, 49% after), opioids (30% before, 51% after), and potassium (22% before, 31% after). Almost half of the patients (49%) used at least one high-risk drug in the 4 months following move-in that they had not used in the four months prior. The most frequently initiated high-risk drugs were opioids (28%), potassium (17%), and anticoagulants and platelet inhibitors (15%). In contrast, 24% of patients used at least one high-risk drug in the 4 months before move-in that they no longer used in the 4 months after, with the most frequent being anticoagulants and platelet inhibitors (12%), potassium (7.4%), and opioids (7.0%) (Supplementary Table 6). Restricting the study cohort to patients who survived the 4 months after move-in did not affect the results of the analysis on high-risk drug use (data not shown).

**Fig. 2:**
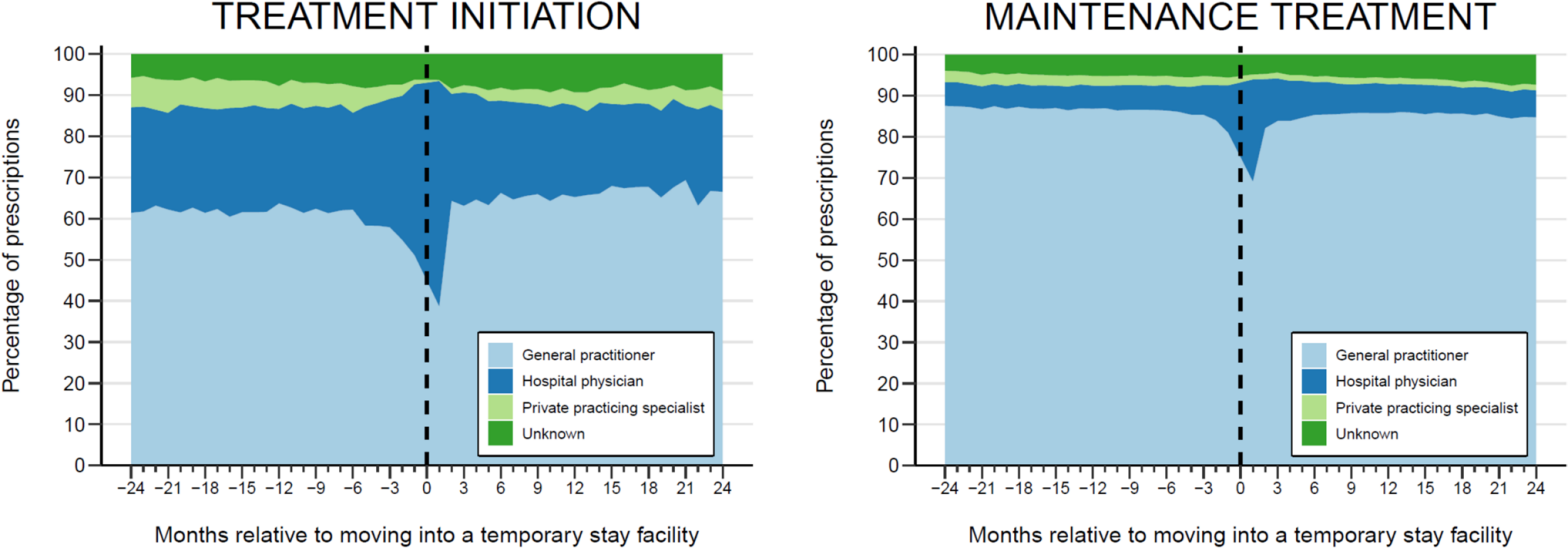
Monthly distribution of prescriber types responsible for initiating treatments (incident prescriptions, left panel) and maintaining treatments (nonincident prescriptions, right panel) in the 2 years before and after moving into a temporary stay facility

GPs were responsible for most treatment initiations prior to move-in (62% 6 months prior to move-in), followed by hospital physicians and private practicing specialists (24% and 6.5%, respectively, 6 months prior to move-in). This pattern remained stable until 6 months prior to move-in when hospital physicians began issuing a growing share of incident prescriptions, while GPs and private practicing specialists saw a decrease. In the month following move-in, hospital physicians were responsible for the majority of incident prescriptions (55%), with GPs accounting for 39%. By two months after move-in, the distribution of prescriber types had returned to levels similar to those before move-in, although with a slight increase in the proportion of incident prescriptions issued by GPs and a decrease in those issued by hospital physicians and private practicing specialists (Fig. 2).

For maintenance treatment, GPs issued 80–90% of nonincident prescriptions, though this proportion dropped to 69% in the month after move-in. During this period, hospital physicians’ share of maintenance prescriptions rose to 25%, while private practicing specialists’ share decreased to 1.2%.

## Discussion

In this drug utilisation study, we describe medication use among patients in temporary stays in Denmark. We found a high level of polypharmacy, with half of the patients using at least six drug classes at the time of move-in. Treatment initiations increased sharply around move-in and remained slightly elevated thereafter. Patients had a substantial use of high-risk drugs, which increased after move-in. GPs were the primary prescribers for both initiating and maintaining treatments, although hospital physicians played a crucial role in initiating new treatments around move-in.

The primary strength of this study is the use of a nationwide registry with high-quality prescription fill data [14, 19], ensuring comprehensive patient coverage and reducing the potential for selection bias. An additional advantage is that the registry captures filled rather than issued prescriptions, eliminating the impact of primary nonadherence, when patients fail to fill an initial prescription for a new drug [20].

A limitation of the study is that it relies on prescription fill data, so we cannot be certain that patients took the drugs as prescribed. However, a recent Danish study found that most patients (68%) who moved into temporary stays after hospital discharge received help with medication management before hospitalization [12]. Additionally, healthcare staff at Danish temporary stay facilities provide support with medication management [8], minimizing the risk of nonadherence impacting on our results. Another limitation is that some drugs can be purchased over the counter, which are not recorded in the Danish National Prescription Registry [14]. However, as many patients in temporary stays receive help with medication management, which typically involves prescribed medications, this limitation may be mitigated. Further, because the Danish National Prescription Registry does not capture drugs used during hospital stays [14], medications initiated during hospital admissions may appear as new prescriptions after move-in, potentially inflating the initiation rate following move-in. Finally, our choice of using the Danish Patient Safety Authority’s list of high-risk drugs is largely arbitrary, as there is, to our knowledge, no international consensus on the definition of high-risk drugs. This list excludes drugs that are known to carry high risks for older adults, such as antipsychotics, benzodiazepines, and non-steroidal anti-inflammatory drugs [21–23], and includes drugs that are generally considered safe, such as dipeptidyl peptidase-4 inhibitors, a class of antidiabetics [24]. The appropriateness of some of the included high-risk drugs depends on the individual patient’s health status, where avoiding or discontinuing the drug, when it is well-indicated, may itself be inappropriate. For example, oral anticoagulants remain underutilised in older adults with atrial fibrillation, despite their increased risk of stroke [25, 26]. However, based on the available data, we were unable to determine whether (changes in) the patients’ use of drugs, including high-risk drugs, reflected good clinical practice or not. Although conceptually different from the assessment of high-risk drugs, we could have examined the patients’ use of potentially inappropriate medications using lists such as the STOPP [21], STOPPFrail [22], and Beers Criteria [23].

This study is the first to systematically describe medication use among patients in temporary stays across Denmark. A recent study on a smaller cohort of patients moving into a temporary stay facility after hospital discharge reported a median of eight drugs at move-in [12]. This is higher than the median in our study, possibly due to differences in the populations studied (a single temporary stay facility versus nationwide data) or because their results were based on issued rather than filled prescriptions. This may also explain their higher reported use of opioids (47% versus 30%) and digoxin (14% versus 5.6%). The substantially increased use of high-risk drugs reported in their study (96% versus 70%) is likely explained by their broader definition of risk drugs, which included frequently used medications such as beta blockers and angiotensin-converting enzyme (ACE) inhibitors or angiotensin II receptor blockers. Interestingly, the medication use of patients in temporary stays closely mirrors that of Danish care home residents, both in terms of number and types of drugs used as well as medication changes [24]. To our knowledge, only few international studies have described patients in temporary stays, none of which provide detailed information about their medication use [27–30].

We observed a marked increase in treatment initiations around move-in, with laxatives, analgesics, and antibiotics being the most frequently initiated drug classes. This finding aligns with previous research on Danish care home residents [31], suggesting that the health decline leading to a temporary stay often results in increased medical interventions, including hospitalisation and GP visits. Increased medical attention likely contributes to medication changes, including the initiation of drugs previously purchased over the counter, such as laxatives. Notably, 86% of patients moving into a temporary stay facility after hospital discharge have at least one drug initiated during their hospital stay [12]. Although we observed a substantial increase in prescriptions initiated by hospital physicians around move-in, GPs continued to play a considerable role in initiating new treatments [32].

Our findings can be used to inform the optimisation of medication use and management in temporary stay facilities. The extensive use of drugs, including high-risk drugs, and frequent medication changes suggest that these patients could benefit from structured medication reviews upon move-in or later during their stay to ensure a better and safer use of drugs. Screening tools such as the STOPP/START [21], STOPPFrail [22], and Beers Criteria [23] may help identify potentially inappropriate medications. Patients in temporary stays are generally older adults with multimorbidity and polypharmacy, which increases their risk of side effects and interactions. While some drugs, such as opioids, may be necessary, others, such as cholesterol-lowering drugs, may cause more harm than benefit. For older patients with frailty and limited life expectancy, the time to benefit of some drugs may exceed their life expectancy [33, 34]. This underscores the importance of individualised medication reviews to align treatment plans with the patient’s current health status and care goals. Alternatively, medication reviews could be conducted in general practice after the patients leave the temporary stay facilities and return home or to a care home, where they are more likely to be medically stable.

Patients in temporary stays are particularly vulnerable to adverse drug events due to medication errors, which can have serious consequences. Therefore, it is essential that transitions to temporary stay facilities as well as the facilities themselves are organised in a way that minimises uncertainty about the patient’s medication. An example of an effort to facilitate safer transitions from hospital to municipal care in Denmark is the recently implemented “72-h extended treatment responsibility”, where the discharging hospital department remains responsible for a patient’s treatment for 72 h after discharge to municipal care, provided they were hospitalised for at least 24 h. During this period, municipal healthcare professionals can consult the discharging hospital for medical advice or guidance. Evaluations of this agreement showed that most calls to discharging hospital departments come from temporary stay facilities and concern medication-related issues and that it has strengthened cross-sectional communication and improved treatment quality and safety [35, 36]. In continuation of this, temporary stay facilities could benefit from having a consultant physician (or possibly a pharmacist) physically present on-site to help provide an overview of and optimise the patient's medication (e.g., through medication reviews), quickly address any medication-related issues, and support the professional development of care staff responsible for medication management. This practice has been implemented in some temporary stay facilities in Denmark, but far from all [8].

Our findings also underscore the seriousness of the recognized issue that, for patients discharged from hospitals, temporary stay facilities only receive a less detailed discharge notice (a type of care plan), while the full discharge letter is sent to the patient’s general practitioner, who has the overall responsibility for the patient’s treatment. The discharge notice does not always include information about the patient's health status or current medication. Further, the Shared Medication Record, which contains information about active medication and prescriptions for all Danish citizens and is used by care staff at temporary stay facilities to get an overview of the patient’s medication, is not always updated [8, 12]. A recent study found discrepancies between the discharge notice and letter in 83% of cases, mostly related to medication. Nearly one-third (31%) of cases where discrepancies required action from the care staff, such as contacting the discharging hospital or the patient’s general practitioner, could have been avoided if the temporary stay facility had received the discharge letter [12]. Uncertainty about these patients’ complex, high-risk, and rapidly changing medication can compromise their safety and be time-consuming for care staff. Providing temporary stay facilities with the discharge letter, which details the patient’s health status and current medication (including any recent changes), could potentially improve patient safety and streamline the medication management process.

In conclusion, we found a high level of medication use among patients in temporary stays, with a marked increase in new treatment initiations around move-in. The use of high-risk drugs also increased after move-in, underscoring the need for careful medication management in these settings. Our findings provide valuable insights that can help guide efforts to optimize medication use and management in temporary stay facilities, ultimately improving patient safety and quality of life.

## Supplementary Information

Below is the link to the electronic supplementary material.

Supplementary file1 (DOCX 32 kb)

## Data Availability

Data on temporary stays were obtained after agreement with the individual Danish municipalities, and the national health registry data were used under license from the Danish Health Data Authority. Therefore, data cannot be made publicly available.
