# Supplementary material for: Medication use patterns among older patients in temporary stays in Denmark
Source: Eur Geriatr Med. 2025 May 7;16(4):1427–35. doi: 10.1007/s41999-025-01210-2 (PMC12378489; doi:10.1007/s41999-025-01210-2)
Supplement: Supplementary file 1 — Supplementary file1 (DOCX 32 kb) [file 41999_2025_1210_MOESM1_ESM.docx]

**Supplementary information**

**Title: Medication use patterns among older patients in temporary stays in Denmark**

**Journal:** European Geriatric Medicine

**Authors:** Hanin Harbi, Carina Lundby, Peter Bjødstrup Jensen, Søren Post Larsen, Linda Grouleff Rørbæk, Lene Vestergaard Ravn-Nielsen, Jesper Ryg, Mette Reilev, Kasper Edwards, Anton Pottegård

**Corresponding author:** Anton Pottegård, Clinical Pharmacology, Pharmacy and Environmental Medicine, Department of Public Health, University of Southern Denmark, Campusvej 55, 5230 Odense M, Denmark. Tel.: +45 28 91 33 40. Email: apottegaard@health.sdu.dk

**Supplementary Table 1**

Baseline characteristics of patients moving into temporary stay facilities in 14 Danish municipalities from 2016 to 2023

|  | Total |
| --- | --- |
|  | (n = 11,424) |
| Sex |  |
| Female | 6,141 (54%) |
| Male | 5,283 (46%) |
| Age |  |
| Median (IQR) | 81 (73-87) |
| < 75 years | 3,394 (30%) |
| 75-84 years | 4,017 (35%) |
| ≥ 85 years | 4,013 (35%) |
| Charlson Comorbidity Index (CCI) |  |
| Median (IQR) | 1 (0-2) |
| 0-1 | 5,752 (50%) |
| 2-3 | 3,921 (34%) |
| ≥ 4 | 1,751 (15%) |
| Hospitalizations in the year before move-in |  |
| Median (IQR) | 3 (2-6) |
| 0-2 | 4,209 (37%) |
| 3-5 | 3,939 (34%) |
| ≥ 6 | 3,276 (29%) |

**Supplementary Table 2**

Use of main drug groups, defined by the first level of the Anatomical Therapeutic Chemical (ATC) code, at time of moving into a temporary stay facility ^a^

| ATC code | Main drug group | Proportion of prevalent users, n (%) |
| --- | --- | --- |
|  |  | (n = 11,424) |
| N | Nervous system | 8,240 (72) |
| C | Cardiovascular system | 8,077 (71) |
| A | Alimentary tract and metabolism | 6,702 (59) |
| B | Blood and blood-forming organs | 5,779 (51) |
| J | Antiinfectives for systemic use | 3,848 (34) |
| R | Respiratory system | 2,797 (24) |
| M | Musculoskeletal system | 2,477 (22) |
| H | Systemic hormonal preparations, excl. sex hormones and insulins | 1,881 (16) |
| G | Genito urinary system and sex hormones | 1,823 (16) |
| D | Dermatologicals | 1,498 (13) |
| S | Sensory organs | 1,479 (13) |
| P | Antiparasitic products, insecticides and repellents | 333 (2.9) |
| L | Antineoplastic and immunomodulating agents | 152 (1.3) |
| V | Various | 24 (0.21) |

^a^Medication use at the day of moving into the temporary stay facility was determined by assessing filled prescriptions within four months prior to move-in.

**Supplementary Table 3**

Top 25 most frequently initiated drug classes during the peak incidence rate observed around move-in (i.e., from five months before to four months after move-in)

| ATC code | Drug class | Proportion of incident users, n (%) |
| --- | --- | --- |
|  |  | (n = 11,424) |
| A06AD | Osmotically acting laxatives | 3,526 (31) |
| N02AA | Natural opium alkaloids | 3,262 (29) |
| N02BE | Anilides | 2,750 (24) |
| A06AB | Contact laxatives | 2,658 (23) |
| J01CA | Penicillins with extended spectrum | 2,462 (22) |
| A12BA | Potassium | 1,955 (17) |
| A02BC | Proton pump inhibitors | 1,891 (17) |
| C03CA | Sulfonamides, plain | 1,399 (12) |
| J01CE | Beta-lactamase sensitive penicillins | 1,241 (11) |
| B01AF | Direct factor Xa inhibitors | 1,188 (10) |
| N05CF | Benzodiazepine related drugs | 1,103 (9.7) |
| N02AX | Other opioids | 1,053 (9.2) |
| D01AC | Imidazole and triazole derivatives | 1,047 (9.2) |
| A02AA | Magnesium compounds | 1,028 (9.0) |
| J01CF | Beta-lactamase resistant penicillins | 979 (8.6) |
| B01AC | Platelet aggregation inhibitors excl. heparin | 820 (7.2) |
| N06AX | Other antidepressants | 813 (7.1) |
| H02AB | Glucocorticoids | 810 (7.1) |
| J01CR | Combinations of penicillins, incl. beta-lactamase inhibitors | 779 (6.8) |
| A03FA | Propulsives | 756 (6.6) |
| N02BF | Gabapentinoids | 755 (6.6) |
| N05BA | Benzodiazepine derivatives | 750 (6.6) |
| N06AB | Selective serotonin reuptake inhibitors | 736 (6.4) |
| J01MA | Fluoroquinolones | 722 (6.3) |
| M01AE | Propionic acid derivatives | 675 (5.9) |

**Supplementary Table 4**

Top 25 most frequently initiated drug classes during the peak incidence rate in the premove-in phase (i.e., the five months before move-in)

| ATC code | Drug class | Proportion of incident users, n (%) |
| --- | --- | --- |
|  |  | (n = 11,424) |
| N02AA | Natural opium alkaloids | 1,090 (9.5) |
| J01CA | Penicillins with extended spectrum | 909 (8.0) |
| N02BE | Anilides | 899 (7.9) |
| A06AD | Osmotically acting laxatives | 775 (6.8) |
| A12BA | Potassium | 633 (5.5) |
| A02BC | Proton pump inhibitors | 620 (5.4) |
| J01CE | Beta-lactamase sensitive penicillins | 614 (5.4) |
| A06AB | Contact laxatives | 548 (4.8) |
| N02AX | Other opioids | 537 (4.7) |
| C03CA | Sulfonamides, plain | 488 (4.3) |
| H02AB | Glucocorticoids | 425 (3.7) |
| J01CF | Beta-lactamase resistant penicillins | 412 (3.6) |
| M01AE | Propionic acid derivatives | 404 (3.5) |
| B01AF | Direct factor Xa inhibitors | 367 (3.2) |
| N02BF | Gabapentinoids | 338 (3.0) |
| J01CR | Combinations of penicillins, incl. beta-lactamase inhibitors | 336 (2.9) |
| D01AC | Imidazole and triazole derivatives | 333 (2.9) |
| A02AA | Magnesium compounds | 325 (2.8) |
| N05CF | Benzodiazepine related drugs | 308 (2.7) |
| A03FA | Propulsives | 304 (2.7) |
| N06AX | Other antidepressants | 290 (2.5) |
| J01MA | Fluoroquinolones | 286 (2.5) |
| N06AB | Selective serotonin reuptake inhibitors | 283 (2.5) |
| B01AC | Platelet aggregation inhibitors excl. heparin | 251 (2.2) |
| N05BA | Benzodiazepine derivatives | 232 (2.0) |

**Supplementary Table 5**

Top 25 most frequently initiated drug classes during the peak incidence rate in the postmove-in phase (i.e., the four months after move-in)

| ATC code | Drug class | Proportion of incident users, n (%) |
| --- | --- | --- |
|  |  | (n = 11,424) |
| A06AD | Osmotically acting laxatives | 2,751 (24) |
| N02AA | Natural opium alkaloids | 2,172 (19) |
| A06AB | Contact laxatives | 2,110 (18) |
| N02BE | Anilides | 1,851 (16) |
| J01CA | Penicillins with extended spectrum | 1,553 (14) |
| A12BA | Potassium | 1,322 (12) |
| A02BC | Proton pump inhibitors | 1,271 (11) |
| C03CA | Sulfonamides, plain | 911 (8.0) |
| B01AF | Direct factor Xa inhibitors | 821 (7.2) |
| N05CF | Benzodiazepine related drugs | 795 (7.0) |
| D01AC | Imidazole and triazole derivatives | 714 (6.2) |
| A02AA | Magnesium compounds | 703 (6.2) |
| J01CE | Beta-lactamase sensitive penicillins | 627 (5.5) |
| B01AC | Platelet aggregation inhibitors excl. heparin | 569 (5.0) |
| J01CF | Beta-lactamase resistant penicillins | 567 (5.0) |
| N06AX | Other antidepressants | 523 (4.6) |
| N05BA | Benzodiazepine derivatives | 518 (4.5) |
| N02AX | Other opioids | 516 (4.5) |
| N06AB | Selective serotonin reuptake inhibitors | 453 (4.0) |
| A03FA | Propulsives | 452 (4.0) |
| J01CR | Combinations of penicillins, incl. beta-lactamase inhibitors | 443 (3.9) |
| J01MA | Fluoroquinolones | 436 (3.8) |
| N05AD | Butyrophenone derivatives | 426 (3.7) |
| N02BF | Gabapentinoids | 417 (3.7) |
| A06AG | Enemas | 414 (3.6) |

**Supplementary Table 6**

Proportion of patients (n = 11,424) filling at least one prescription for a high-risk drug in the four months before and after moving into a temporary stay facility

|  | 4 months before move-in, n (%) | 4 months after move-in, n (%) | Only during the 4 months before move-in, n (%) | Only during the 4 months after move-in, n (%) | During both the 4 months before and after move-in, n (%) |
| --- | --- | --- | --- | --- | --- |
| Anticoagulants and platelet inhibitors | 5,245 (46) | 5,627 (49) | 1,333 (12) | 1,715 (15) | 3,912 (34) |
| Antidiabetics | 1,831 (16) | 1,760 (15) | 374 (3.3) | 303 (2.7) | 1,457 (13) |
| Digoxin | 641 (5.6) | 750 (6.6) | 210 (1.8) | 319 (2.8) | 431 (3.8) |
| Low-dose methotrexate | 80 (0.70) | 68 (0.60) | 37 (0.32) | 25 (0.22) | 43 (0.38) |
| Opioids | 3,425 (30) | 5,794 (51) | 804 (7.0) | 3,173 (28) | 2,621 (23) |
| Potassium | 2,466 (22) | 3,523 (31) | 851 (7.4) | 1,908 (17) | 1,615 (14) |
| Any | 7,949 (70) | 9,434 (83) | 2,729 (24) | 5,612 (49) | 6,600 (58) |
